# Experimental study on the influence of locked-in stress on the uniaxial compressive strength and elastic modulus of rocks

**DOI:** 10.1038/s41598-020-74556-1

**Published:** 2020-10-15

**Authors:** Xin Liu, Hansheng Geng, Hongfa Xu, Yinhao Yang, Linjian Ma, Lu Dong

**Affiliations:** State Key Laboratory of Disaster Prevention & Mitigation of Explosion & Impact, Army Engineering University of PLA, Nanjing, 210007 China

**Keywords:** Civil engineering, Structural materials

## Abstract

The rock contains many inclusions which produce high locked-in stress under the ground stress. In order to study the influence of locked-in stress on the mechanical properties of rocks, the rock-like materials and nitrile rubber particles are used to make a test block of the rock-like model which contains inclusions. The rubber particles will expand as the test block is heated, which creates locked-in stress in the inclusions. Uniaxial compression tests of similar model blocks with different locked-in stresses and different inclusion contents were performed by using a water bath and MTS-5T uniaxial compression testing machine. The results show that the peak strength and elastic modulus decreased with the increasement of locked-in stress and inclusion content. In the meantime, the relationship among the peak strength, the elastic modulus of the test piece, the locked-in stress and the inclusion content were obtained with the help of a mathematical fitting analysis of the quantitative formula. Furthermore, the expression and value curve of the joint impact factor are calculated. This paper evaluates the importance of the locked-in stress in the mechanical properties of the rock-like material and provide a guide for other researchers to further investigate the locked-in stress in rocks.

## Introduction

**Figure 1:**
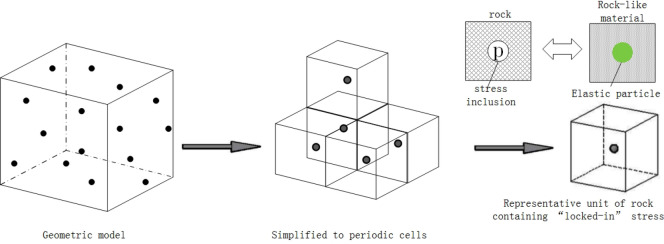
Representative unit of rock containing locked-in stress.

Rocks are heterogeneous media which consist of solid mineral, cement between mineral particles and micro-pores as shown in Fig. 1. However, there are fluid or other types of media existing in micro-pores as well^1^. The randomness of rock composition has a significant impact on the mechanical properties of the rock. In the past, the problems of mechanical analysis, numerical simulation of rock mechanics and geotechnical engineering were often analyzed through the continuum mechanics of a macroscopic perspective. Therefore, the influence of micro-anisotropy of rock materials was ignored. Nowadays, the development of underground space has reached an unprecedented depth, where the mechanical behavior of rock mass is different from that of the shallow rock mass due to higher stress, temperature, and hydraulic pressure. In a stressful environment, many disasters in rock engineering are difficult to be explained and predicted, such as the rock-burst, and the uplift of the roadway floor, etc. This research presents the rock mechanics in an environment of the deep earth to reevaluate the influence of the “locked-in” stress on the mechanical properties of the rock.

In 1974, Muller put forward the locked-in stress concept^2^. Tan suggested the hypothesis of the cause of locked-in stress in rock^3^. In the locked-in stress theory, the tectonic loading and the thermal loading make incompatible strain in the rock which results in a non-uniform inter-granular stress field. Some of these stresses are loosened due to the dislocation movement and micro rupture, and the other part of stresses are still retaining in rock and are called the “sealed” stress. The sealed stress is called stress inclusions and there are many stress inclusions in rock. This kind of stress will be released when the external disturbance reaches a certain point, or the rock itself undergoes creep. The phenomenon of locked-in stress being released is often encountered in practice. For example, the abnormal upheaval of the bottom of excavation far more than expected from gravity influence. Moreover, the occurrence of rock-bursts in tunnels is also the result from the release of strained energy and it may also play a role in earthquakes^4^. Over the years, this situation has not been investigated sufficiently. Recently, some scholars refocus on this field. Qian et al. proposed the problem of nonconforming deformation of micro-pores and studied the influence of the density and length of micro-cracks on self-balanced residual stress and rock mass failure^5–7^. Wang studied the mechanism of rock-burst taking into account the release of “locked-in”, in which the stress inclusion is considered as the friction between particles due to non-coordinated deformation of rock^8^. Yue demonstrated the pressure and volume expansion of the fluid inclusions. Further researches proposes the possibility of micro-fluid inclusions which enclosed that intact rock may be in a compressed state and have a high pressure and it can cause local exceptionally high self-balancing stress field inside the rock which can be destroyed during the excavation process and resulting in the rock-burst in local surrounding rock^9,10^.

The locked-in stress exists in the original rock environment and the self-balance of locked-in stress can be easily changed or broken even disappear due to the release of underground stress and the artificial disturbance of sampling process. Therefore, it is difficult to directly sample the rock containing locked-in stress and study its mechanical mechanism. The secondary difficulty in this study is the stress in rock, which cannot be quantitatively measured.

In the fields of rock mechanics, rock-like materials are usually utilized according to the similar theory of study. Based on the mechanism of internal stress in rocks, (Eshelby’s inclusion theory) and the differences of the thermal expansion coefficients of various materials^11^, the locked-in stress with different temperature fields of inclusion model were intended to simulate. By this mean, the intrinsic stress can be generated inside the inclusions. Nitrile-butadiene rubber (NBR) is selected as an inclusion material, followed by the thermal expansion coefficient and elastic modulus of NBR at different temperatures. In order to select appropriate rock-like material^12^, the influence was deduced of temperature and elastic parameters of rock-like material on the simulated locked-in stress and the identified appropriate rock-like material. After that, the locked-in stress can be obtained by controlling the production and maintenance temperature of the sample and adjusting the ambient temperature of the sample after 28 day’s maintenance. In this research, uniaxial compression tests were performed on specimens with different levels of inclusions and various values of locked-in stress. The influence of the locked-in stress and inclusion contents on the macroscopic mechanical properties of the rock are obtained by mathematical analysis of the experimental data.

## Determination of the properties of inclusion

The shape and distribution of closed pores in the rock are random, as shown in Fig. 1. The assumptions of this research are summarized:

1. The stress inclusions in the rock are spherical and randomly distributed in the rock matrix.
2. The volume fraction and size of the locked-in stress inclusions are kept smaller to avoid interaction between the inclusions.

Based on the above two assumptions, a geometrical model of rock with stress inclusions is constructed by a cube rock representative unit (RVE) with a single spherical stress inclusion in the geometric center^13^. To simulate the rock and inclusion material, the high elastic material with characteristics of rock-like material as well as high thermal expansion coefficient is proposed with the geometrical model and thermal stress theory. This paper presents the fabrication of similar material, containing locked-in stress and quantitative method for the stress value from the experimental point of view.

The representative unit in Fig. 1 is the inclusion model of the equivalent inclusions’ theory. In order to make the locked-in stress (MPa) in the rock-like matrix, the intrinsic stresses with different temperatures (°C) are applied based on the difference in the thermal expansion coefficients between the inclusion material and rock-like matrix.

Eshelby proposed a theoretical solution for the problem of a single ellipsoidal inclusion in an infinite interior in the elastic field and proposed the equivalent inclusions theory^11^. One important conclusion is that the elastic strain field inside the ellipsoid particles, when the volume fraction of the inclusions is small, is uniform when the intrinsic strain of the inclusions is uniform. It can be expressed as:

1 $$ \varepsilon_{kl}^{^{\prime}} = S_{ijmn} \varepsilon_{mn}^{*} $$

where $\varepsilon_{mn}^{*}$ is the permanent deformation of inclusions, i.e., phase change or intrinsic strain, in case of no matrix constraint; $\varepsilon_{kl}^{^{\prime}}$ is the disturbance strain, caused by different mechanical properties; $S_{ijmn}$ is the fourth order Eshelby tensor, related to the shape of the inclusions and the Poisson's ratio of the matrix.

The eign-strain of the thermal expansion mismatch is:

2 $$ \varepsilon_{kl}^{*} = \delta_{kl} (\alpha_{1} - \alpha_{0} )\Delta T $$

where $\varepsilon_{kl}^{*}$ is the eign-strain;$\alpha_{0}$ and $\alpha_{1}$ is the linear expansion coefficient of the matrix and the inclusion; $\Delta T$ is the temperature change value; $\delta_{ij}$ is Kronecker symbol.

For an ellipsoidal inhomogeneous inclusion with an eign-strain $\varepsilon_{ij}^{*}$, it can be replaced by a homogeneous inclusion with the shape and the different intrinsic strain $\varepsilon_{ij}^{*1}$ from $\varepsilon_{ij}^{*}$. Its equivalent equation is expressed as:

3 $$ C_{ijkl}^{1} {(}\varepsilon_{kl}^{^{\prime}} { - }\varepsilon_{kl}^{*} {) = }C_{ijkl}^{0} {(}\varepsilon_{kl}^{^{\prime}} { - }\varepsilon_{kl}^{*1} {)} $$

where $C_{ijkl}^{0}$ and $C_{ijkl}^{1}$ are elastic constants of the matrix phase and the inclusion phase, respectively, and have:

4 $$ C_{ijkl} = \lambda \delta_{ij} \delta_{kl} + 2G\delta_{ik} \delta_{jl} $$

where $\lambda$ and $G$ are the first order and second order of the Lame constants.

According to the inclusion theory, the temperature and the locked-in stress of the inclusion under ideal conditions can be obtained from a series of deductions. In the case of small deformation, the stress along each direction of the points is equal, and the force of the inclusion of the matrix is normal stress which is perpendicular to the spherical contact surface. The magnitude of the stress is:

5 $$ \sigma_{L} = \frac{{2E_{1} E_{0} (\alpha_{1} - \alpha_{0} )\Delta T}}{{\mu_{0} E_{1} - 4\mu_{1} E_{0} + 2E_{0} + E_{1} }} $$

The equation of locked-in stress expressed by shear modulus and bulk modulus:

6 $$ \sigma_{L} = \frac{{(\alpha_{1} - \alpha_{0} )}}{{\frac{1}{{4G_{0} }} + \frac{1}{{3K_{1} }}}}(T - T_{0} ) = (\alpha_{1} - \alpha_{0} )\frac{{12G_{0} K_{1} }}{{3K_{1} + 4G_{0} }}(T - T_{0} ) $$

where *p* is the pressure between the particle inclusion and the matrix after the change of the temperature field; $E_{0}$, $\mu_{0}$,$E_{1}$ and $\mu_{1}$ are the elastic modulus and Poisson's ratio of the matrix material and the inclusion material, respectively; $T_{0}$ is the temperature at which the stress is zero, here $T_{0}$ is equal to 16.5 °C; *G*_0_ is the shear modulus of matrix material; *K*_1_ is the bulk modulus of the inclusion material. This method is only suitable for the simulations of the pressure of a spherical inclusion. When the rubber particles are in ordinary ellipsoids, the thermal expansion does not uniform, hence, the stress on the surface of the rubber particles is also non-uniform. Moreover, when the pressure of the fluid in the pores remains constant, then there is no similar relationship between them.

**Figure 2:**
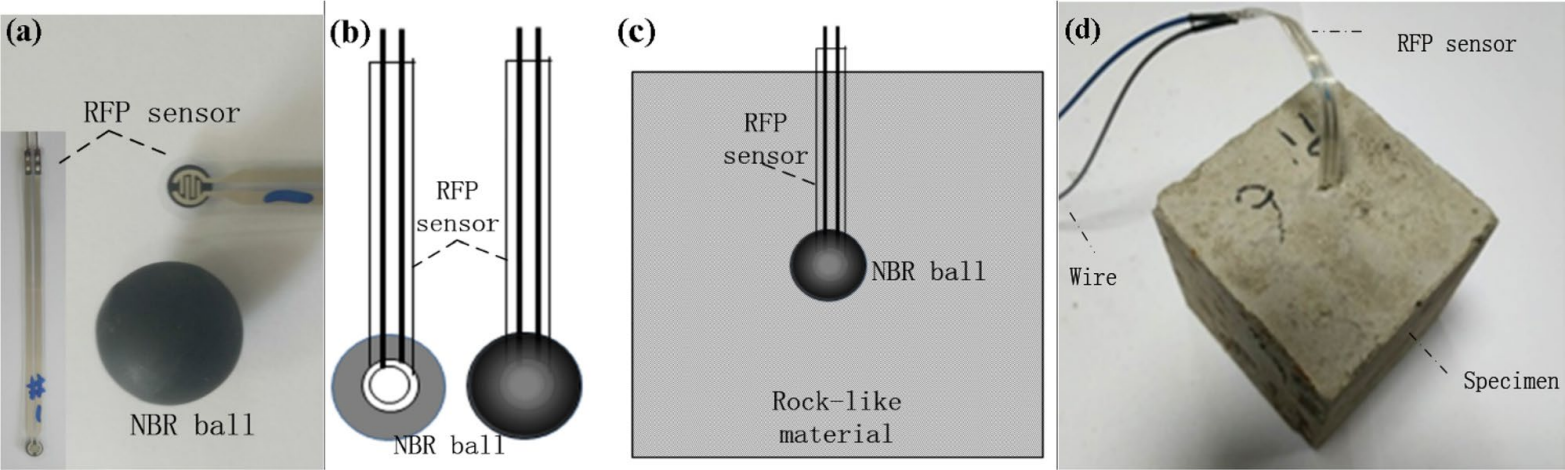
Measure the locked-in stress in the inclusion by film pressure sensor. (**a**) Rubber ball before embedding the sensor, (**b**) rubber ball after embedding the sensor, (**c**) put into the matrix slurry and (**d**) specimen of locked-in stress.

**Figure 3:**
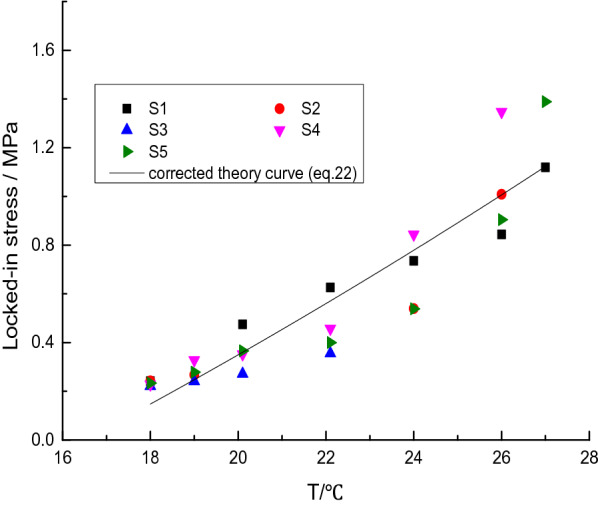
Testing results of locked-in stress.

Equation (6) is the ideal solution and it cannot predict the actual situation due to the changing conditions of the environmental, material and conservation in the deformation and other factors, the results will produce some errors. In order to fix these errors, a test method for the relatively accurate relationship of locked-in stress with the temperature. Placing the membrane pressure sensor in the spherical inclusions then its pressure sensor is poured into rock-like material samples, as shown in Fig. 2. To be specific, RFP sensor represents resistive film pressure sensor, NBR represents nitrile-butadiene rubber. Since the inclusions are spherical that the force generated by the intrinsic strain is the hydrostatic pressure. Therefore, the film pressure sensor can be used to measure the confining stress. The corresponding locked-in stress by changing temperature can be measured. The specific result is shown in Fig. 3. The detailed experimental tests and theoretical calculations can be seen in literature^14^.

According to the measurement of locked-in stress, Eq. (6) is modified as:

7 $$ \sigma_{L} = 9.622 \times 10^{ - 4} (T + 79.56)(T - 16.5) $$

## Test schemes

### Selection of material

The mechanical properties of rocks through rock-like materials have been extensively studied. Typical rock-like materials are cement mortar, gypsum-cement mixed mortar, epoxy resin mortar, urea–formaldehyde resin mortar and sand-phenolic resin. The latest achievements in rock-like materials include: NIOS materials (Tsinghua University), MIB materials (Wuhan University), MSB materials. Brace used flat glass to study the rock damage evolution at uniaxial compression^15^; Bobet et al. studied the initiation of rock fractures with gypsum as rock-like material^16,17^. Zhu proposed the usage of resin to make a transparent rock-like material^18^. On the other hand, Zhang et al. investigated the failure mode of rock under the coupling effect of the spherical explosion and static by cement mortar^19^.

The thermal expansion coefficient of geo-materials is in order of 10^−5^–10^−6^/°C in the range of 10–60 °C. The principle of selection of inclusions is to make the thermal expansion coefficient of inclusion large enough that the thermal expansion of the rock-like material can be ignored. So, the thermal expansion coefficient of the inclusion material is preferably larger than 1 × 10^−4^ × /°C. The thermal expansion coefficient of metal materials is in the range of 1–2 × 10^−6^/°C^20^. The thermal expansion coefficient of the inorganic materials is generally in the range of 10^−5^–10^−6^/°C^21^. The thermal expansion coefficient of polymer materials is generally higher, while the linear expansion coefficient of NBR, celluloid and polyamide is in the order of 1 × 10^−4^. NBR has excellent elasticity, and its elastic modulus and shear modulus are negligible compared to the common rock. So, it can be approximated as fluid. Moreover, NBR is selected to make the spherical inclusion due to the features of ubiquitous and easy-to-shape. In this section, the mechanical properties of NBR were experimentally studied.

As a kind of rock material, the elastic modulus and strength of cement mortar can be adjusted on a large scale. Based on the previous studies, this paper employs cement mortar as the rock-like material matrix. The material is made of water, 325 Portland cement and quartz sand with the ratio of 0.7:1:3.

**Figure 4:**
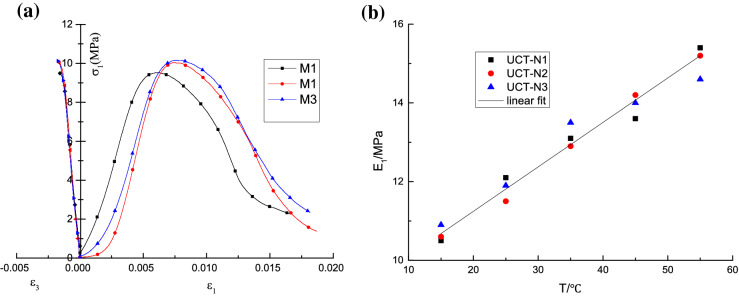
Test results of each component of the sample. **(a**) Uniaxial compressive stress–strain curves for matrix material and (**b**) temperature-modulus of elastic relationship of NBR.

The uniaxial compressive stress–strain curves of rock-like matrix specimens were obtained from laboratory tests, shown in Fig. 4a. The relationship between the elastic modulus of nitrile rubber and temperature is shown in Fig. 4b. Where, M1, M2 and M3 are the rock-like matrix sample numbers; UCT-N1, UCT-N2 and UCT-N3 are the rubber sample numbers.

The data is compared as shown in Fig. 4a and the mechanical parameters of the rock-like substrate samples are listed in Table 1. In Table 1, the peak strength of the rock-like matrix is the maximum axial stress on the stress–strain curve and the elastic modulus is the slope of the linear segment, near half the peak strength on the stress–strain curves.

**Table 1:** The mechanical parameters of the matrix material sample.

Sample no	Elastic modulus (MPa)	Peak strength (MPa)	Poisson's ratio
M1	2.44	9.52	0.28
M2	2.82	10.04	0.33
M3	2.41	10.16	0.29
Average value	2.56	9.91	0.30

From the linear regression analysis of the experimental data of Fig. 4b, the elastic modulus of nitrile rubber is obtained as follows:

8 $$ E_{{1}} { = 0}{\text{.000113}}T + 0.00899 $$

where: *E*_1_ is the Elastic modulus of nitrile rubber (MPa); *T* represents temperature (°C).

According to the literature^22^, the change of Poisson's ratio of nitrile rubber with temperature is not obvious. It is usually 0.49.

### Test scheme and sample production

In order to test the rock-like block’s mechanics properties at different locked-in stress and different inclusion contents, four experimental schemes were planned. Each scheme considers one locked-in stress with four different levels of inclusions, as shown in Table 2. Where *ρ* represents inclusion content.

**Table 2:** Test schemes.

Scheme no	$\sigma_{L}$ (MPa)	*T* (°C)	Sample no			
			*ρ* (%)			
			1	2	3	4
I	0	16.5	S00-01	S00-02	S00-03	S00-04
II	0.4	20.6	S04-01	S04-02	S04-03	S04-04
III	0.8	24.5	S08-01	S08-02	S08-03	S08-04
IV	1.2	28.0	S12-01	S12-02	S12-03	S12-04

As can be seen from Table 2, the locked-in stresses are 0, 0.4, 0.8 and 1.2 MPa while the contents of inclusions are 1, 2, 3 and 4% respectively. From Eq. (7), the temperature values for each scheme can be calculated, which are 16.5, 20.6, 24.5 and 28.0 °C respectively.

As described above, a specific proportion of rock-like matrix is used. Spherical rubber particles are added during the process of stirring to evenly distribute the rubber particles in the cement mortar. After that, the slurry was poured into cylindrical molds with the size of $\Phi 50\;{\text{mm}} \times 100\;{\text{mm}}$. Then the sample was put into the thermostatic chamber for 28 days of conservation.

### Uniaxial compression test

For the uniaxial compression test, the sample should be waterproof and is heated in a thermostat (water bath) to the corresponding temperature of locked-in stress. The heating time should be more than 30 min. After that the uniaxial compression test can be performed.

**Figure 5:**
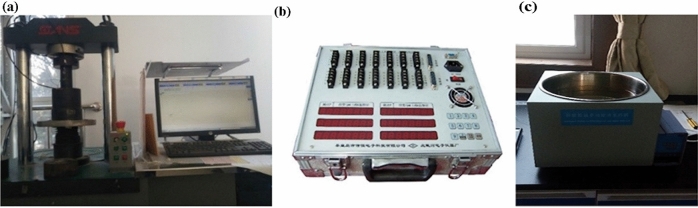
Test equipment. (**a**) MTS-5Tcompression testing machine, (**b**) strain gauge and (**c**) thermostat water bath.

The instruments used in this experiment are shown in Fig. 5: intelligent digital multi-functional oil–water bath heating pot; strain gauge of Qinhuangdao Longke Measurement and Control Technology Co., Ltd; MTS 5T uniaxial-compression testing machine. The uniaxial compression speed was set to 0.2 mm/min and the acquisition cycle of strain gage is 0.5 s.

## Test results

**Figure 6:**
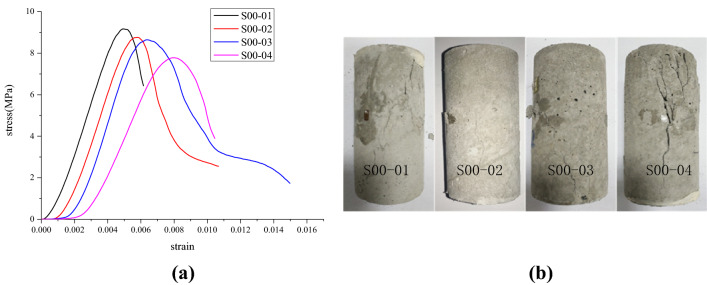
Test results of scheme I (*σ*_*L*_ = 0). (**a**) Stress–strain curves and (**b**) samples after destruction.

**Figure 7:**
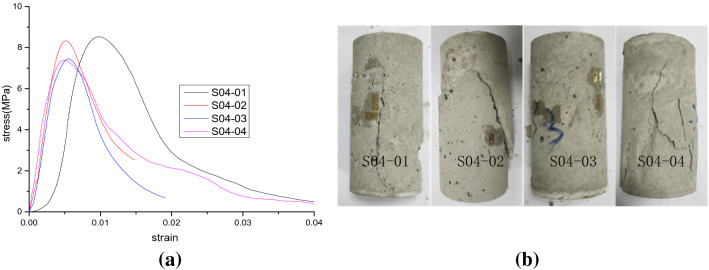
Test results of scheme II (*σ*_*L*_ = 0.4 MPa). (**a**) Stress–strain curves and (**b**) samples after destruction.

**Figure 8:**
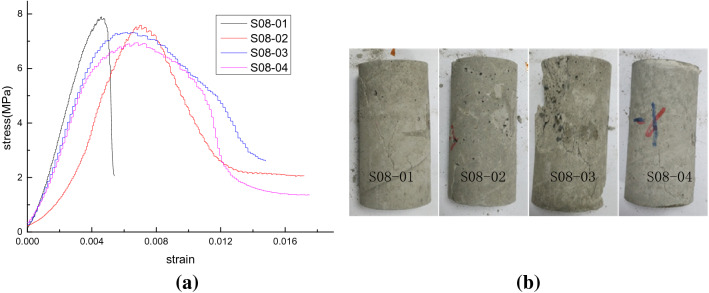
Test results of scheme III (*σ*_*L*_ = 0.8 MPa). (**a**) Stress–strain curves and (**b**) samples after destruction.

**Figure 9:**
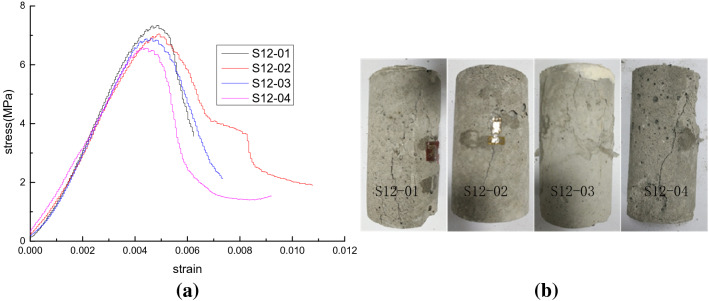
Test results of scheme IV (*σ*_*L*_ = 1.2 MPa). (**a**) Stress–strain curves and (**b**) samples after destruction.

The stress–strain curves of the four test blocks with the locked-in stress of 0, 0.3, 0.8, and 1.2 MPa are shown in Figs. .6, 7, 8 and 9, respectively. The vertical axis denotes the values of the axial stress while the horizontal axis represents the corresponding axial strain. Figure 6 shows the uniaxial compressive stress–strain curves of four different kinds of sample contents at the locked-in stress of 0 MPa. Furthermore, the uniaxial compressive stress–strain curve of four different samples at 0.4 MPa of locked-in stress is shown in Fig. 7. Figure 8 shows the uniaxial compressive stress–strain curves of four different samples at the Locked-in stress of 0.8 MPa. Finally, Fig. 9 shows the uniaxial compressive stress–strain curves of four different samples at the locked-in stress of 1.2 MPa. In addition, the damage of the sample in the test is mainly shear failure it can be seen in Figs. 6, 7, 8 and 9. Meanwhile, with the increase of the inclusion contents, the number of microcracks when the sample is broken increases.

According to the stress–strain curves in Figs. 6, 7, 8 and 9, the elastic modulus and the peak strength of each sample can be obtained. The peak strength is obtained from the maximum axial stress on the stress–strain curve while the elastic modulus is the slope of the linear segment near half the peak strength on the stress–strain curve. The peak strength and elastic modulus of each test sample are shown in Table 3. *E* represents the modulus of elasticity; $\sigma_{c}$ represents the peak strength.

**Table 3:** Peak strength and elastic modulus of test samples.

Scheme	Sample no	*ρ* (%)	$\sigma_{L}$ (MPa)	*E* (GPa)	Decrease rate of *E* (%)	$\sigma_{c}$ (MPa)	Decrease rate of $\sigma_{c}$ (%)	Peak strain
I	S00-01	1	0	2.51	2.0	9.26	6.6	0.0049
	S00-02	2	0	2.47	3.5	8.77	11.5	0.0058
	S00-03	3	0	2.41	5.9	8.36	15.6	0.0063
	S00-04	4	0	2.36	7.8	7.89	20.4	0.0080
II	S04-01	1	0.4	2.40	6.3	8.52	14.0	0.0057
	S04-02	2	0.4	2.30	10.2	8.33	15.9	0.0032
	S04-03	3	0.4	2.23	12.9	7.75	21.8	0.0052
	S04-04	4	0.4	2.16	15.6	7.38	25.5	0.0036
III	S08-01	1	0.8	2.11	17.6	7.89	20.4	0.0048
	S08-02	2	0.8	2.03	20.7	7.59	23.4	0.0072
	S08-03	3	0.8	2.00	21.9	7.32	26.1	0.0061
	S08-04	4	0.8	1.88	26.6	6.95	29.9	0.0068
IV	S12-01	1	1.2	1.98	22.7	7.34	25.9	0.0048
	S12-02	2	1.2	1.92	25.0	7.05	28.9	0.0048
	S12-03	3	1.2	1.89	26.2	6.89	30.5	0.0044
	S12-04	4	1.2	1.79	30.1	6.58	33.6	0.0044

## Discussion and analysis

It can be demonstrated as shown in Table 3 that the inclusion content and the value of locked-in stress strongly affected the mechanical properties of rocks. The main findings can be summarized from the following three aspects.

1. With the increment of inclusions, the peak strength and elastic modulus of the samples decrease gradually. As shown in Scheme II (*σ*_*L*_ = 0.4 MPa), when the inclusion content increases from 0 to 4%, the peak strength of the sample decreases by 14.0, 15.9, 21.8, and 25.5% respectively, compared to the peak intensity of the matrix. The elastic modulus of the sample is 6.3, 10.2, 12.9, and 15.6% lower than the elastic modulus of the matrix, respectively.
2. With the increment of locked-in stress, the peak strength and elastic modulus of the samples decrease gradually. For example, when the inclusion content is 2%, the Locked-in stress increases from 0 to 1.2 MPa and the peak strength of the sample decreases by 11.5, 15.9, 23.4, and 28.9% respectively, compared to the peak strength of the matrix. The elastic modulus of the sample is 3.5, 10.2, 20.7, and 25.0% respectively lower than the elastic modulus of the matrix.
3. In terms of the factor of efficiency that the effect of locked-in stress and inclusion contents on the peak strength and elastic modulus of the test sample are combined. With the decrease of the inclusion contents, the influence of the locked-in stress on the peak strength and the elastic modulus decreases gradually. When the content of the inclusion is zero, the effect of locked-in stress also disappeared while the peak strength and elastic modulus of the sample are the peak strength and elastic modulus of the matrix. This can be obtained from the average value of Table 1. Therefore, a group of data (the peak strength equals to 9.91 MPa and the elastic modulus equals to 2.56 GPa) at zero content is added to each scheme of this study.

To reflect the generality of the test results, the test data are treated in a dimensionless manner. The specific peak strength, specific modulus of elasticity and specific Locked-in stress are rearranged as follows:

9 $$ \left. {\begin{array}{*{20}l} {\overline{\sigma }_{{\text{c}}} = \frac{{\sigma_{c} }}{{\sigma_{c0} }}} \hfill \\ {\overline{E} = \frac{E}{{E_{0} }}} \hfill \\ {\overline{\sigma }_{L} = \frac{{\sigma_{L} }}{{\sigma_{c0} }}} \hfill \\ \end{array} } \right\} $$

where $\overline{\sigma }_{{\text{c}}}$ represents the specific peak strength; $\sigma_{c}$ represents the peak strength; $\sigma_{c0}$ represents the peak strength of matrix material; $\overline{E}$ represents the specific modulus of elasticity; $E$ represents the modulus of elasticity; $E_{0}$ represents the modulus of elasticity of matrix material; $\overline{\sigma }_{L}$ represents specific locked-in stress and $\sigma_{L}$ represents locked-in stress.

**Figure 10:**
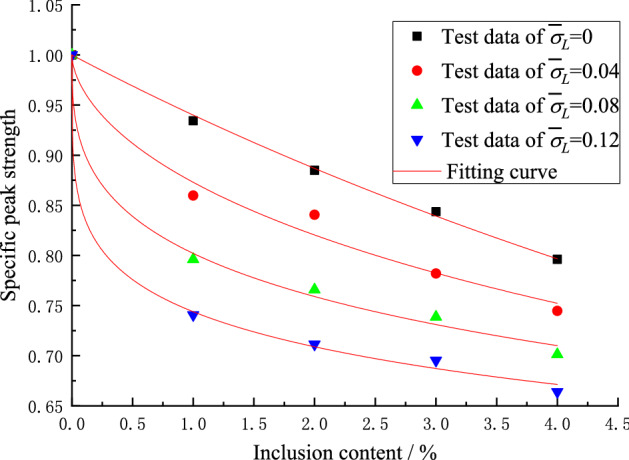
Influence of specific locked-in stress and inclusion contents on specific peak strength.

**Figure 11:**
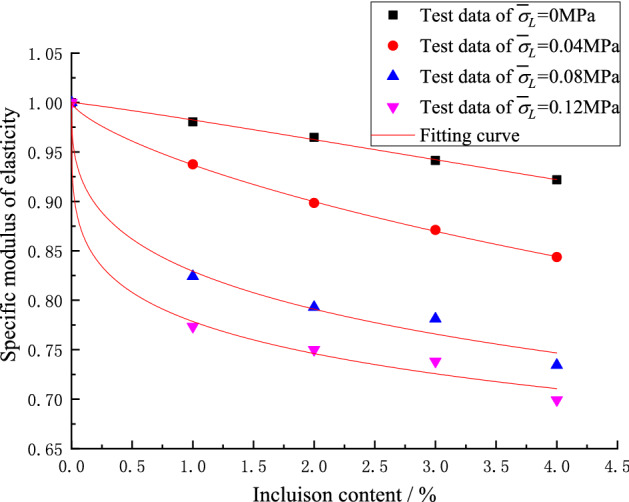
Influence of specific locked-in stress and inclusion contents on specific modulus of elasticity.

The test data of Table 3 is plotted in Figs.10 and 11 which show the effect of the locked-in stress and the inclusion contents on the peak strength and the elastic modulus, respectively.

After the analysis of the test data, the formula (10) can be derived to well express the change of specific peak strength and specific elastic modulus with the inclusion contents.

10 $$ \left. {\begin{array}{*{20}l} {\overline{\sigma }_{{\text{c}}} = \frac{1}{{1 + m\rho^{n} }}} \hfill \\ {\overline{E} = \frac{{1}}{{1 + a\rho^{b} }}} \hfill \\ \end{array} } \right\} $$

where *ρ* represents inclusion content; *m*, *n*, *a*, *b* are the parameters which are going to be determined and be changed with the locked-in stress, respectively.

The test data in Figs. 10 and 11 (corresponding to the four different closure stresses) are non-linearly fitted by Eq. (10), the values of *m*, *n*, *a*, *b* are obtained and listed in Table 4. The fitting curves are shown by the solid lines in Figs. 10 and 11.

**Table 4:** Fitting parameters.

Scheme	$\overline{\sigma }_{L}$	Fitting parameters for specific peak strength			Fitting parameters for specific modulus of elasticity		
		*m*	*n*	Correlation coefficient *R*^2^	*a*	*b*	Correlation coefficient *R*^2^
I	0	6.38188	1	0.99787	3.10556	1.11924	0.99659
II	0.04036	2.19282	0.58907	0.97775	1.92579	0.72851	0.99965
III	0.08073	1.31438	0.36319	0.99475	1.08239	0.36056	0.98658
IV	0.12109	1.10888	0.25397	0.99756	0.93697	0.25891	0.99226

**Figure 12:**
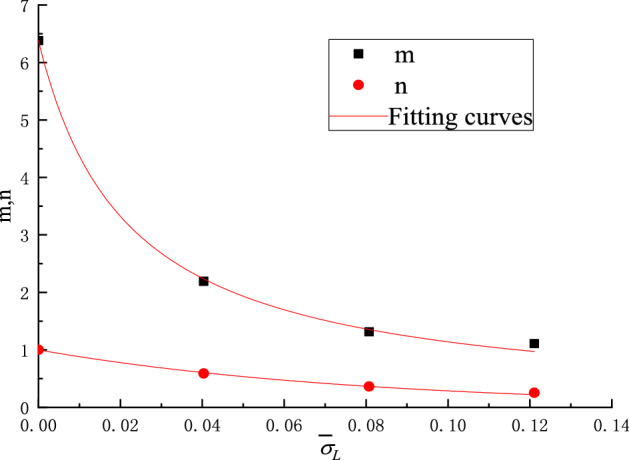
Relationship between *m*, *n* and specific locked-in stress.

**Figure 13:**
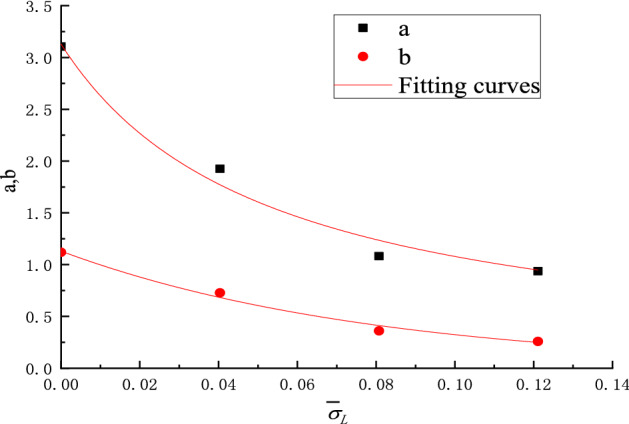
Relationship between *a*, *b* and specific locked-in stress.

Table 4 presents the quantitative curve fitting results where it can be seen that the correlation coefficients are close to 1. Therefore, Eq. (10) can be used to predicts the changing of specific peak strength as well as the specific elastic modulus with the content of inclusions. It can be seen from Table 4 that the fitting parameters *m*, *n*, *a*, *b* change with the change of the specific locked-in stress and the changing trends can be easily observed from Figs. 12 and 13.

The following relational equations can be easily obtained by mathematical fitting analysis of the parameters of *m*, *n*, *a*, *b* in Figs. 12 and 13:

11 $$ \left. {\begin{array}{*{20}l} m = \frac{6.38}{{1 + 46\overline{\sigma }_{L} }}&\quad {(}R^{2} { = }0.99879) \hfill \\ n = \exp ( - 12.{5}\overline{\sigma }_{L} )&\quad {(}R^{2} { = }0.99583) \hfill \\ a = \frac{3.13}{{1 + 19\overline{\sigma }_{L} }}&\quad {(}R^{2} { = }0.98389) \hfill \\ b = 1.13\exp ( - 12.5\overline{\sigma }_{L} )&\quad {(}R^{2} { = }0.98910) \hfill \\ \end{array} } \right\} $$

By substituting (11) into (10), the mathematical expression of specific peak intensity and specific modulus can be obtained as follows:

12 $$ \left. {\begin{array}{*{20}l} {\overline{\sigma }_{c} = \frac{{1 + 46\overline{\sigma }_{L} }}{{1 + 46\overline{\sigma }_{L} + 6.38\rho^{{\exp ( - 12.5\overline{\sigma }_{L} )}} }}{ = }\frac{{1 + 46\overline{\sigma }_{L} }}{{1 + 46\overline{\sigma }_{L} + 6.38\kappa }} \, } \hfill \\ {\overline{E} = \frac{{1 + 19\overline{\sigma }_{L} }}{{1 + {19}\overline{\sigma }_{L} + 3.13\left[ {\rho^{{\exp ( - 12.5\overline{\sigma }_{L} )}} } \right]^{1.13} }}{ = }\frac{{1 + 19\overline{\sigma }_{L} }}{{1 + {19}\overline{\sigma }_{L} + 3.13\kappa^{1.13} }} \, } \hfill \\ \end{array} } \right\} $$

where

13 $$ \kappa { = }\rho^{{\exp ( - 12.5\overline{\sigma }_{L} )}} $$

**Figure 14:**
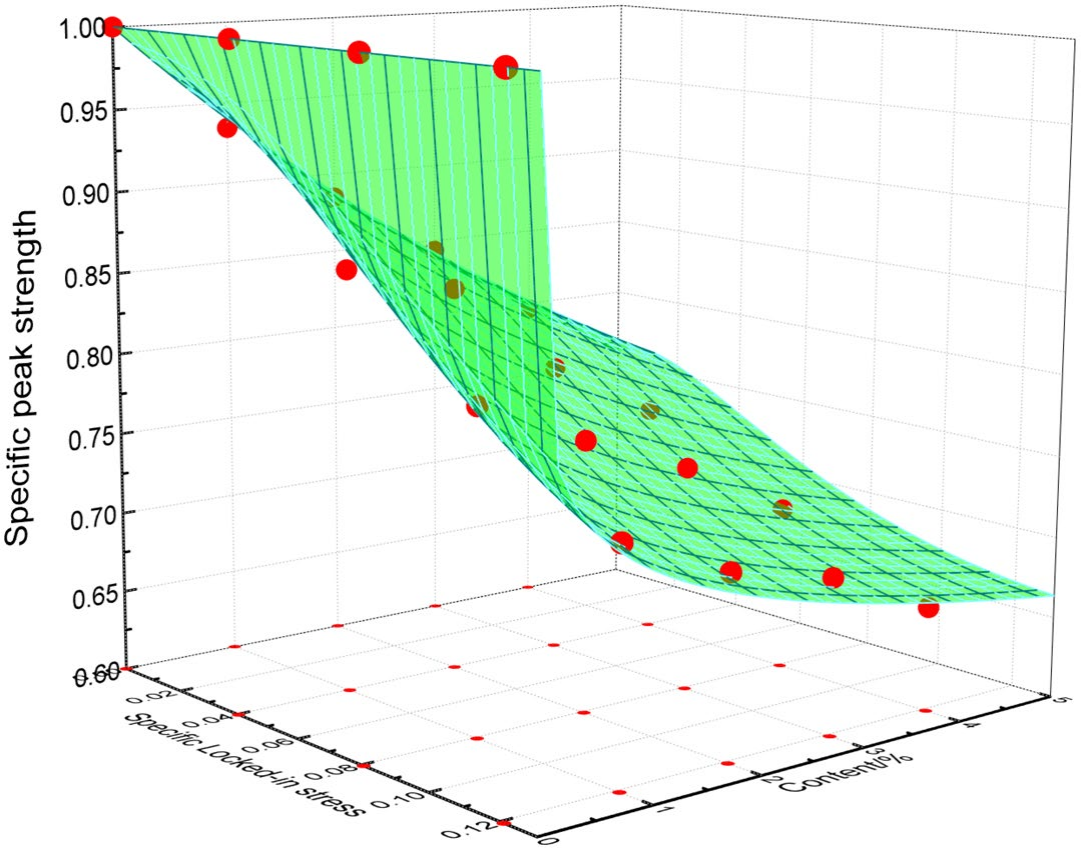
Comparison between mathematical results and test data of specific peak strength.

**Figure 15:**
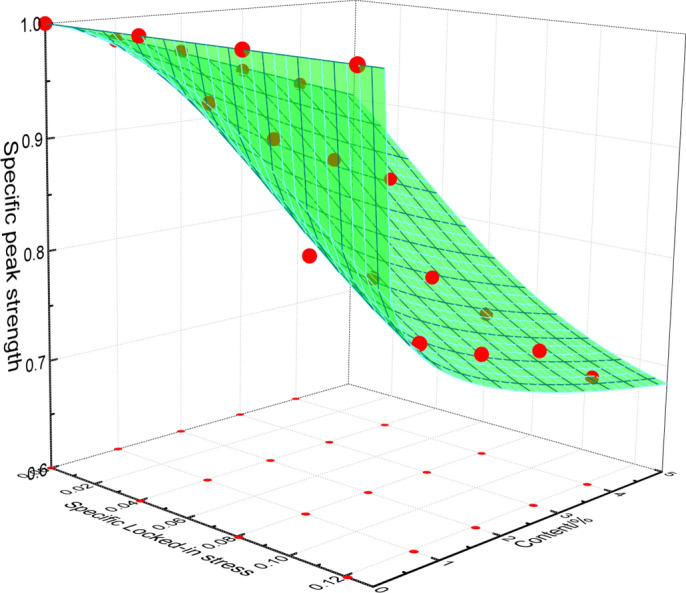
Comparison between mathematical results and test data of modulus of elastic.

The relationship of strength and elastic modulus of rocks under uniaxial compressive with locked-in stress can be easily estimated with the help of Eq. (12). Figures 14 and 15 compare the experimental data with the calculated results using Eq. (12). It can be concluded that the proposed mathematical model can well predict the evolution trend of rock strength and elastic modulus. The maximum error between the experimental value of the strength and the calculated value of the mathematical model is 2.1%, and the maximum error of the experimental value of the elastic modulus and the calculated value of the mathematical model is 3%.

From Eq. (12) that the strength and elastic modulus of the rock-like block have a close relationship with the locked-in stress and the inclusion contents. Moreover, the impact of joint impact factor *κ* is greater among these parameters. It should be pointed out that the mathematical model of Eq. (12) is only suitable for the samples with inclusions of small content and the specific locked-in stress ratio should be smaller, generally *ρ* < 0.1, and $\overline{\sigma }_{L}$ < 0.2.

**Figure 16:**
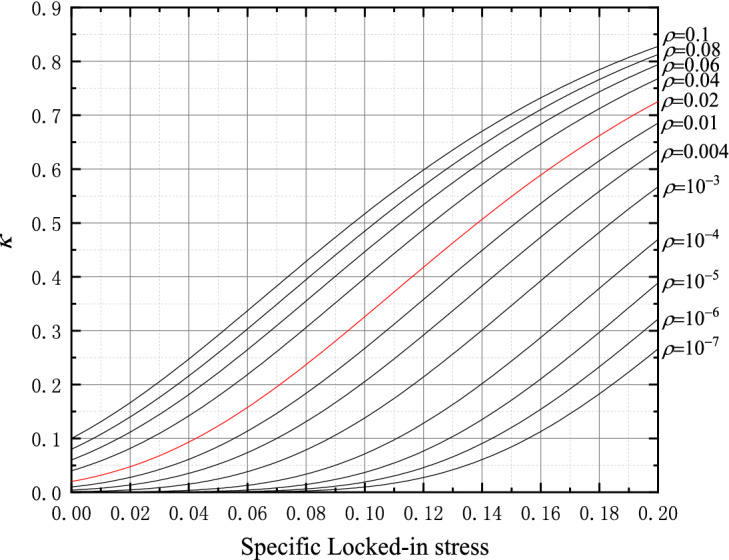
Impact factor *κ.*

The curves of the impact factor *κ* can be drawn with Eq. (13), as shown in Fig. 16 and it is easy to estimate the value of the impact factor *κ*. Even though the content of inclusions is small, the impact factor *κ* is relatively large because the locked-in stress can be large. It is further demonstrated that the influence of the locked-in stress on the mechanical properties of the rock cannot be ignored for the deeply underground rocks with a small inclusion contents, which might be subjected to a large locked-in stress.

## Conclusion

Inclusions are one of the most important geologic features of rocks. Large locked-in stress resulted from long-term ground stress released after excavation and will have a great impact on the stability of underground rock. Rock blocks containing locked-in stress cannot be sampled in situ because the removed block will gradually release its locked-in stress when it leaves the original geologic environment (stressed environment). Cement-based materials and rubber particles are utilized in this study to produce rock-like blocks model and uniaxial compression tests of rock-like blocks were performed with a water bath and MTS-5T uniaxial compression testing machine. Conclusions can be summarized as follows:

1. By heating the test block of rock-like material, the locked-in stress of inclusion in the rock-like block is simulated with the thermal expansion of the rubber particles. Based on the above principle, a similar rock test piece can be made easily. Hence, it is feasible to use this method to investigate the influences of the locked-in stress on the mechanical rocks.
2. The stress–strain curves of each sample with different amounts of locked-in stress and inclusion content were obtained by heating and performing uniaxial compression of the rock-like samples. These curves reflect the mechanical characteristics of the test blocks under different conditions. It is found that the peak strength and elastic modulus of the rock blocks decrease with the increase of the locked-in stress and the inclusion contents. The effect of locked-in stress on the peak strength and elastic modulus gradually decreases. When the inclusion content is zero, the locked-in stress has no effect on the peak strength and elastic modulus due to the loss of power carrier of locked-in stress.
3. Mathematically, a quantitative formula is obtained to predict the peak strength and elastic modulus with varying locked-in stress and inclusion contents. This analysis has pointed out the mathematical expression and the curve of the joint impact factor.
